# Cell collectivity regulation within migrating cell cluster during Kupffer's vesicle formation in zebrafish

**DOI:** 10.3389/fcell.2015.00027

**Published:** 2015-05-07

**Authors:** Takaaki Matsui, Hiroshi Ishikawa, Yasumasa Bessho

**Affiliations:** Gene Regulation Research, Nara Institute of Science and TechnologyNara, Japan

**Keywords:** collective cell migration, cell adhesion, Kupffer's vesicle, cell signaling, zebrafish

## Abstract

Although cell adhesion is thought to fasten cells tightly, cells that adhere to each other can migrate directionally. This group behavior, called “collective cell migration,” is observed during normal development, wound healing, and cancer invasion. Loss-of-function of cell adhesion molecules in several model systems of collective cell migration results in delay or inhibition of migration of cell groups but does not lead to dissociation of the cell groups, suggesting that mechanisms of cells staying assembled as a single cell cluster, termed as “cell collectivity,” remain largely unknown. During the formation of Kupffer's vesicle (KV, an organ of laterality in zebrafish), KV progenitors form a cluster and migrate together toward the vegetal pole. Importantly, in this model system of collective cell migration, knockdown of cell adhesion molecules or signal components leads to failure of cell collectivity. In this review, we summarize recent findings in cell collectivity regulation during collective migration of KV progenitor cells and describe our current understanding of how cell collectivity is regulated during collective cell migration.

## Introduction

Individual cells have the potential to migrate randomly. When chemo-attractants are distributed in an environment, the cells can migrate toward the attractants. In some situations, cell aggregates can move directionally while maintaining cell adhesions in a process called collective cell migration. Collective cell migration is essential for the generation of basic organ structures such as sheets, clusters, spheres, sprouts, and vesicles in the morphogenetic processes of animal development (Rorth, 2009, 2012; Weijer, 2009; Reig et al., 2014), and is also observed in wound closure and cancer invasion (Friedl and Gilmour, 2009; Friedl et al., 2012). Collective cell migration shares some features with individual cell migration but also has unique ones. These features have been described in several in-depth reviews cited in this review (Friedl and Gilmour, 2009; Rorth, 2009, 2012; Weijer, 2009; Friedl et al., 2012; Reig et al., 2014).

Loss-of-function of chemo-attractants, their receptors, signal mediators, and cell adhesion molecules blocks or delays the directed migration of the cell groups in model systems of collective cell migration such as zebrafish lateral lines and *Drosophila* border cells (Niewiadomska et al., 1999; Kerstetter et al., 2004; Wilson et al., 2007; Friedl and Gilmour, 2009; Rorth, 2009; Reig et al., 2014). However, these manipulations unexpectedly does not dissociate the cell groups, suggesting that how “cell collectivity,” a feature in which cells stay assembled as a single cell cluster, is produced and maintained within a migrating cell cluster remains largely unknown.

Kupffer's vesicle (KV) is a key organ required for the left-right asymmetric patterning in zebrafish (Amack and Yost, 2004; Essner et al., 2005; Matsui and Bessho, 2012). During KV organogenesis, 20–30 KV progenitors called dorsal forerunner cells (DFCs) make a single cluster and migrate together, so KV organogenesis is represented as one of the model systems of collective cell migration. In our and other's studies, loss-of-functional situations of genes/signals have led to a breaking up of the cluster of KV progenitors (DFCs) without affecting their directed migration (Matsui and Bessho, 2012). We therefore feel that KV organogenesis is a good model for investigating the regulatory mechanisms of cell collectivity formation. In this review, we describe our recent understanding of how cell collectivity is generated and maintained during collective DFC migration.

## Review

### KV formation and function

**Figure 1:**
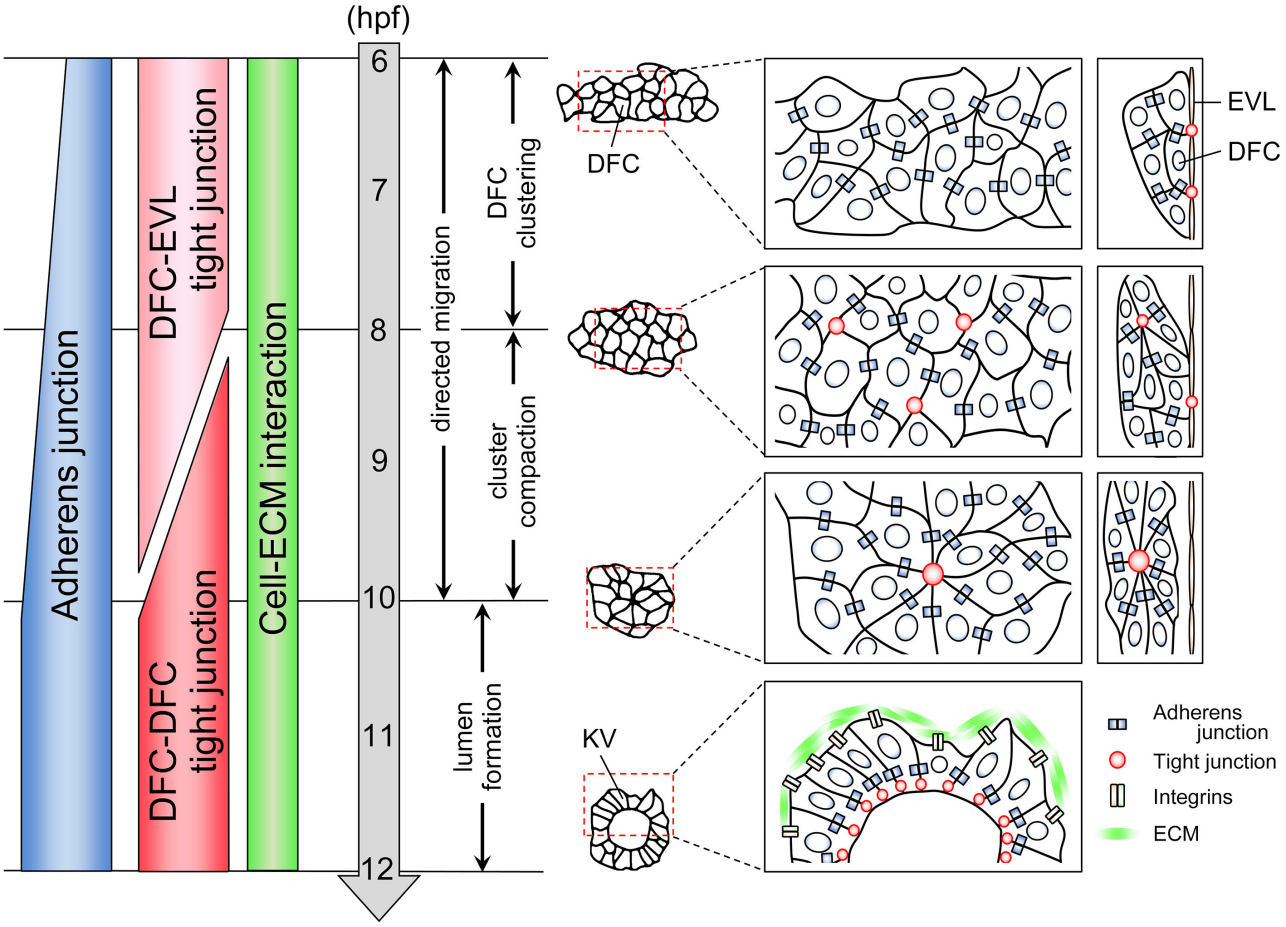
**Cell adhesion status during KV organogenesis**. KV organogenesis is divided into four steps. First, 20–30 DFCs form a cluster through the formation of adherens junction. A subset of DFCs binds to EVL through the formation of tight junction. Second, the cluster becomes compact. Multiple focal points that enrich tight junction components are generated between some DFCs. Third, these points are then rearranged into a single focal point. Fourth, an internal lumen is generated. During collective DFC migration, adherens junction, tight junction, and cell-ECM interaction are generated in a migrating DFC clusters.

In zebrafish, DFCs first appear in the dorsal site adjacent to the embryonic margin at 6 h post-fertilization (hpf) (Figure 1) (Essner et al., 2002, 2005; Oteiza et al., 2008; Matsui and Bessho, 2012). About 20–30 DFCs form a single cluster that migrates toward the vegetal pole accompanied by epiboly movement by 10 hpf (Cooper and D'Amico, 1996; Melby et al., 1996). During migration, the DFC cluster undergoes compaction and changes into a bottled shaped cluster. Around the late-gastrulation stage (8 hpf), DFCs start to polarize so that multiple focal points are generated within the cluster (Cooper and D'Amico, 1996; Melby et al., 1996; Amack et al., 2007). These points are then rearranged into a single focal point that will expand and become a vesicle lumen by 12 hpf. At the same time, motile monocilia are generated on the apical membrane facing the lumen. In the KV, rotation of motile cilia generates a counterclockwise flow of fluid, called nodal flow, and leads to the establishment of left-right asymmetry in the body (Essner et al., 2005; Amack et al., 2007; Oteiza et al., 2008). Several excellent reviews have already described the molecular and cellular mechanisms of left-right patterning and ciliogenesis (Hirokawa et al., 2006, 2012; Ishikawa and Marshall, 2011; Nakamura and Hamada, 2012; Blum et al., 2014; Choksi et al., 2014), so we do not go into those here. Instead, we focus on cell collectivity regulation occurring within a migrating cell cluster.

### Cell adhesive interactions formed in a migrating DFC cluster

When DFCs appear as a single cluster at the mid-gastrulation stage (6 hpf), *cadherin1* and *cadherin2* (E-cadherin and N-cadherin in zebrafish, respectively) are expressed in DFCs (Babb et al., 2001; Babb and Marrs, 2004; Esguerra et al., 2007; Harrington et al., 2007; Warga and Kane, 2007; Matsui et al., 2011), suggesting that cadherin-based adherens junction mediates DFC-DFC interaction (Figure 1). Although, in general, formation of adherens junction leads to generation of tight junction (Hartsock and Nelson, 2008), tight junction is not formed between DFCs at 6 hpf. Instead, tight junction is formed between a subset of DFCs and overlying enveloping layer (EVL) cells (Figure 1) (Oteiza et al., 2008). In addition, it has been reported that *integrinα5* and *integrinβ1b* are expressed in migrating DFC cluster at 6 hpf (Ablooglu et al., 2010). These findings suggest that multiple types of cell adhesions including adherens junctions, tight junctions, and cell-extracellular matrix (ECM) interactions are generated in DFCs at the onset of DFC clustering.

During epiboly (6–10 hpf), *cadherin1* expression increases in migrating DFCs (Figure 1) (Babb et al., 2001; Babb and Marrs, 2004; Esguerra et al., 2007; Matsui et al., 2011), suggesting that a cadherin1-based adherens junction contributes to the compaction and shape change of the cluster. Within the bottled shaped cluster seen in 8–10 hpf, focal points that enrich the tight junction components are generated, and then multiple focal points are rearranged into a single focal point that will be an internal vesicle lumen (Figure 1) (Amack et al., 2007; Oteiza et al., 2008, 2010; Siddiqui et al., 2010). On the other hand, DFC-EVL tight junction is maintained by the end of epiboly (10 hpf) (Figure 1) (Oteiza et al., 2008, 2010; Siddiqui et al., 2010). This means that there is a transition of tight junction formation from DFC-EVL to DFC-DFC pairs during collective DFC migration (Figure 1). However, how the tight junction formation is regulated and how this transition occurs remain unexplored.

### Regulatory mechanisms of cell collectivity formation in the DFC cluster

When *cadherin1* is knocked down specifically in DFCs, the DFC cluster is broken up into multiple groups of cells at 60–80% epiboly stages (6.5–8.5 hpf) (Matsui et al., 2011); the “broken-up DFC cluster phenotype” is represented as a sign of failures of cell collectivity (Table 1). This phenotype eventually results in formation of small KV and randomization of left-right patterning (this means the loss of KV function), indicating that cell collectivity regulation is required for formation of a functional KV. We also find that strengthening a cadherin1-based adherens junction by cadherin1 overexpression induces the formation of a highly compacted oval-shaped DFC cluster, leading to abnormal formation of KV and loss of KV function (Matsui et al., 2011). These observations suggest that KV formation becomes abnormal in either weak or strong cadherin1-based adherens junctions. This may be a common feature of collective migration because it has been reported that both up- and down-regulation of DE-cadherin induce the delayed collective migration of the border cell cluster during *Drosophila* oogenesis (Niewiadomska et al., 1999; Schober et al., 2005).

**Table 1:** **Genes essencial for cell collectivity regulation during collective DFC migration**.

**Loss/Gain of function**	**Gene**	**Protein**	**Adherens junction**	**Tight junction**	**Cell-ECM interaction**	**DFC clustering**	**KV structure**	**References**
DFC-KD	*cadherin1*	Adhesion molecule	Weaken	n.d.	n.d.	Broken-up	Small/disrupted	Esguerra et al., 2007; Oteiza et al., 2010; Matsui et al., 2011
Mutation, KD	*ace/fgf8a*	FGF ligand	Weaken (Reduced cdh1 expression)	n.d.	n.d.	Broken-up	Small/disrupted	Matsui et al., 2011
DFC-KD; KD	*canopy1*	FGF positive regulator	Weaken (Reduced cdh1 expression)	n.d.	n.d.	Broken-up	Small/disrupted	Matsui et al., 2011
DFC-KD; KD	*tbx16*	TF induced by FGF	Weaken (Reduced cdh1 expression)	Weaken (wide aPKC focal points)	n.d.	Broken-up	Small/disrupted	Amack et al., 2007; Matsui et al., 2011
KD	*ier2*	FGF mediator	n.d.	n.d.	n.d.	Broken-up	Small/disrupted	Hong and Dawid, 2009
KD	*fibp1*	FGF mediator	n.d.	n.d.	n.d.	Broken-up	Small/disrupted	Hong and Dawid, 2009
KD	*fgf2*	FGF ligand	n.d.	Normal	n.d.	Broken-up	Small	Arrington et al., 2013
DFC-KD; KD	*syndecan2*	Heparan sulfate proteoglycan	n.d.	Normal	n.d.	Broken-up	Small	Arrington et al., 2013
OE	*gna13a*	Cadherin1 binding G protein	Weaken (Reduced cdh1 binding activity)	n.d.	n.d.	Broken-up	n.d.	Lin et al., 2009
KD	*prickle1a*	nc-Wnt mediator	Weaken (Reduced adhesion forces)	Weaken (wide aPKC focal points)	n.d.	Failed compaction	Fragmented lumens	Oteiza et al., 2010
DFC-KD; KD	*lpar3*	LPA receptor	n.d.	Weaken (Reduced aPKC expression)	n.d.	Broken-up	Small/disrupted	Lai et al., 2012
DFC-KD; KD	*autotaxin*	Lysophospholipase D	n.d.	Weaken (Reduced aPKC expression)	n.d.	Broken-up	Small/disrupted	Lai et al., 2012
DFC-KD; KD	*ttrap*	Nodal antagonist	Weaken (Reduced cdh1 expression)	n.d.	n.d.	Spread	n.d.	Esguerra et al., 2007
OE	*smad3b*	Nodal mediator	Weaken (Reduced cdh1 expression)	n.d.	n.d.	Broken-up	n.d.	Esguerra et al., 2007
KD	*wdr18*	WD-repeat protein	n.d.	Normal	Weaken	Broken-up	Small	Gao et al., 2011
DFC-KD; KD	*integrinα5, β1b*	Adhesion molecule to ECM	n.d.	Normal	Weaken	Broken-up	Small	Ablooglu et al., 2010; Gao et al., 2011
KD	*lgl2*	A homolog of Tumor suppressor protein in Drosophila	Weaken (Reduced cdh1 accumulation at membrane)	Normal	n.d.	n.d.	Small	Tay et al., 2013
DFC-KD; KD	*cateninβ1, β2*	Wnt mediator	n.d.	Normal	n.d.	Small (Reduced proliferation)	Small	Zhang et al., 2012
Inhibition	–	Ca^2+^ -ATPase	Weaken	n.d.	n.d.	Broken-up	Small/disrupted	Schneider et al., 2008

*DFC-KD, DFC-specific knockdown; KD, knockdown; OE, overexpression; n.d., not determined*.

These findings suggest that a fine-tuning of *cadherin1* expression and function into a proper range is required for generating proper DFC collectivity. Our recent study has revealed a part of this mechanism: specifically, we characterized a fibroblast growth factor (FGF) positive feedback regulator *canopy1* in zebrafish and found that FGF-signal dependent regulation of *cadherin1* expression is required for generating proper cell collectivity in the DFC cluster (Matsui et al., 2011). fgf8a secreted from DFCs binds to and activates FGF receptor 1 (fgfr1) in DFCs. The intracellular signal pathway (Ras-Erk pathway) induces *canopy1* expression. canopy1 with chaperones enhances fgfr1 protein folding within endoplasmic reticulum and increases mature fgfr1 at the membrane of DFCs, resulting in much higher FGF/Erk signal activity in DFCs. The potentiated FGF/Erk signal induces the expression of *cadherin1* via transcriptional activation of *tbx16* (Table 1). Furthermore, it has been reported that Nodal signaling regulates the expression of *cadherin1* in DFCs via repression of *snail1* expression (Table 1) (Esguerra et al., 2007), which is known as a transcriptional repressor of E-cadherin expression in several animals (Batlle et al., 2000; Cano et al., 2000; Hajra et al., 2002).

In addition to these transcriptional regulations of *cadherin1*, the post-translational regulations of *cadherin1* contribute to the fine-tuning of cell collectivity. The *lateral giant larvae 2* (*lgl2*), one of the lateral giant larvae genes initially identified as tumor suppressor genes in *Drosophila*, is expressed in DFCs (Tay et al., 2013). lgl2 in cooperation with rab11b GTPase enhances the trafficking of cadherin1 proteins into a lateral membrane to generate KV lumen, suggesting that regulation of the membrane trafficking of cadherin1 protein is required for ensuring cell collectivity (Table 1). Heterotrimeric G proteins of the Gα_12_ family (Gα_12_ and Gα_13_) bind to the intercellular domain of cadherin1 and interfere with the link between cadherin1 and β-catenin, leading to the inhibition of cell-cell adhesion (Lin et al., 2009). Importantly, loss- and gain-of-function of the Gα_12_ family in zebrafish result in a breaking up of the DFC cluster into small groups of cells (Table 1) (Lin et al., 2009), suggesting again that fine-tuning of the *cadherin1* expression is required for generating cell collectivity. Taken together, these results suggest that the cadherin1-based adherens junction is regulated by both transcriptional and post-translational mechanisms and that these fine-tuning systems are essential for generating proper DFC collectivity during collective DFC migration.

The DFC-specific knockdown of *integrinα5* or *integrinβ1b* results in the breaking up of the DFC cluster (Ablooglu et al., 2010), demonstrating that cell-ECM interaction contributes to DFC collectivity (Figure 1 and Table 1). integrinα5/β1b is known to recognize RGD-peptide containing ligands (e.g., fibronectin, vitronectin, and osteopontin). Although overexpression of a dominant negative form of fibronectin and *fibronectin* mutations, including *natter*, leads to the randomization of left-right asymmetric body plan and cardia bifida, respectively (Trinh and Stainier, 2004; Compagnon et al., 2014), it has not been reported that DFC collectivity is compromised in embryos injected with the dominant negative form of fibronectin and *fibronectin* mutants. Although laminin-α1β1γ1 accumulate around KV and DFC-specific knockdown of *laminin-γ1* results in ciliogenesis failures and the randomization of a left-right asymmetric body plan (Compagnon et al., 2014), this manipulation does not lead to the breaking up of the DFC cluster. Therefore, it is unlikely that fibronectin and laminins are a ligand for integrinα5/β1b in migrating DFCs. Additional experiments are required to identify the ligand(s) for integrinα5/β1b during collective DFC migration in the near future.

As stated above, loss-of-function of cadherin1-based adherens junction or integrinα5/β1b-ECM interaction in DFCs leads to a broken up DFC cluster (Table 1) (Ablooglu et al., 2010; Matsui et al., 2011). However, some DFCs bind to each other even in these situations. These findings suggest that cooperation of several adhesive interactions is required to regulate DFC collectivity. Therefore, it will be important to characterize cooperative effects of adhesive interactions on cell collectivity regulation during collective DFC migration.

### Genes essential for DFC collectivity

Many genes have been reported to be involved in the regulation of DFC collectivity, as listed in Table 1. As stated above, genes related to FGF and to Nodal signaling can function as regulators of DFC collectivity. In addition, knockdown of *prickle1a*, a non-canonical Wnt (nc-Wnt) regulator, leads to failure to compact the DFC cluster during late-gastrulation stages (9–10 hpf) (Table 1) (Oteiza et al., 2010), suggesting that nc-Wnt signaling also regulates cell collectivity. This idea is supported by data from the direct single-cell force spectroscopy measurement of adhesion properties between a pair of DFCs, which showed that the adhesion force between DFCs isolated from embryo depleted of *prickle1a* is reduced as compared to wildtype DFCs (Oteiza et al., 2010). This method is very good for measuring cell adhesion forces at the single cell level, while physiological interactions are disrupted during cell preparation. It would be of great interest to develop a method to measure physiological cell adhesion forces within living zebrafish embryos in the near future.

### Identification of genes required for DFC collectivity formation in future

A DFC-specific gene knockdown approach in which morpholino is injected into the yolk of embryos at the 256–512 cell stages has greatly contributed to our understanding of how DFC collectivity is regulated (this review), how DFC clustering occurs and whether DFC/KV morphogenesis is required for the establishment of left-right patterning (Amack and Yost, 2004; Amack et al., 2007; Matsui and Bessho, 2012). We therefore believe that this approach will be important to identify new genes required for DFC collectivity. However, very recently, Kok et al. have reported a problem for morpholino-induced knockdown approach (Kok et al., 2015). Approximately 80% of phenotypes induced by morpholinos are not observed in mutant embryos, indicating that morpholinos highly induce off-target effects; moreover, these problems are shared with other antisense technologies. It is thus recommended that only morpholinos that recapitulate the respective phenotypes seen in mutant embryos should be applied for ancillary analyses. As the DFC-specific gene knockdown approach is based on morpholino-induced knockdown, here is a good opportunity to consider whether we can apply this approach to investigate how DFC collectivity is regulated in future.

As this approach has similarities to the conditional knockout strategy, it is best to evaluate whether DFC-specific gene knockdown recapitulates the phenotypes caused in the DFC-specific conditional knockout zebrafish. Although insertion of loxP site into a zebrafish genome by genome editing technologies such as TALEN and CRISPER/Cas9 systems has recently been successful (Bedell et al., 2012; Chang et al., 2013), a method generating conditional knockout zebrafish has not yet been developed. Even though such a method is likely to be developed in the near future, we feel that it would be difficult to apply it to understand the roles of genes in DFC/KV formation.

In general, conditional gene knockout is carried out by using a Cre/loxP system. The expression of Cre recombinase is induced by a tissue/organ-specific promoter and Cre protein modulates the genome at the specific insertion site of loxP. Thus, it takes time to observe the signs of Cre/loxP recombination. For instance, in the case of conditional knockout mice, the signs of Cre/loxP recombination are detected an average of 24 h after the induction (Hayashi and McMahon, 2002; Chen et al., 2007). In the case of DFCs, however, collective DFC migration starts at almost the same time as the DFC specification (evident by the expression of early DFC marker genes, *sox32* and *sox17*) and finishes just 3 h (Oteiza et al., 2008; Matsui and Bessho, 2012). It has been suggested that Cre/loxP recombination may not occur simultaneously with collective DFC migration without applying much faster recombination methods.

Because the DFC-specific gene knockdown approach is absolutely indispensable to provide new insights into collective DFC migration, KV formation, KV ciliogenesis, and left-right patterning in the near future, a guideline for DFC-specific gene knockdown is needed. We therefore propose one based on guidelines reported by Eisen and Smith (2008) and Kok et al. (2015). First, as with standard morpholino-based knockdown approaches, validate morpholino-induced phenotypes and compare them to those of the mutant. If the mutant is not available, generate one for the gene of interest and make sure of the morpholino-induced phenotypes by comparison. Second, inject the morpholino tagged with fluorescein or lissamine into the yolk of embryos at 256–512 cell stages (2.5–2.75 hpf), and select embryos in which morpholino has been correctly delivered into the DFCs. As an important control, also inject the same morpholino into the yolk of embryos at the sphere-dome stages (4–4.3 hpf) (Amack and Yost, 2004; Amack et al., 2007). Third, perform a rescue experiment by co-injecting morpholino and mRNA into the yolk of embryos at 256–512 cell stages (Matsui and Bessho, 2012).

### Dynamics of collective DFC migration

**Figure 2:**
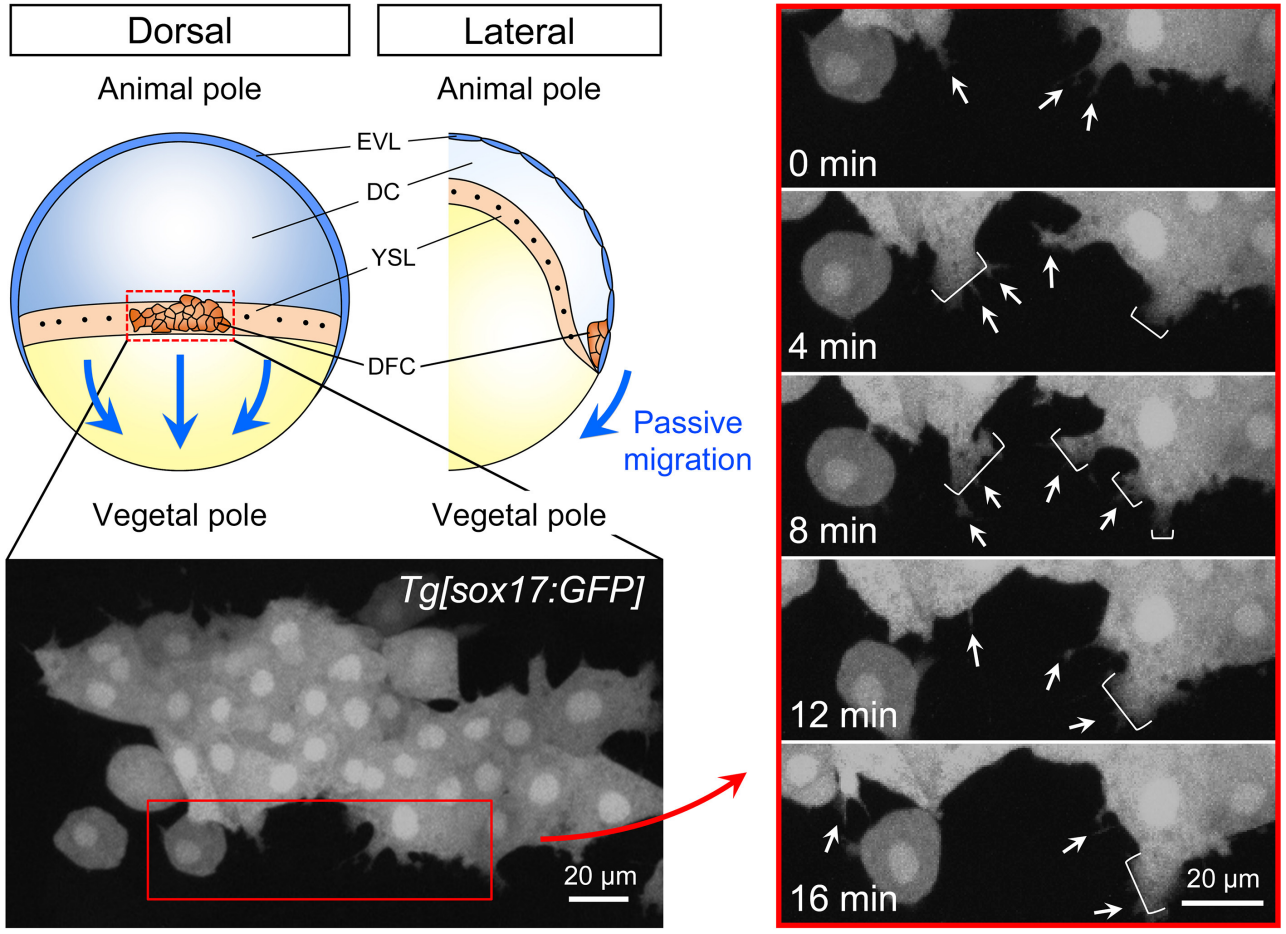
**Cell dynamics during collective DFC migration**. Depending on the formation of tight junction between DFC and EVL, directed migration of the DFC cluster toward the vegetal pole passively occurs (blue arrows). EVL, enveloping layer; DC, deep cells; YSL, yolk syncytial layer; DFC, dorsal forerunner cells. Time-lapse images from 0 to 16 min (4 min intervals) in the control *Tg[sox17:GFP]* embryo (left panels). Dorsal view, anterior to the top. Scale bar: 20 μm. As shown previously (Ablooglu et al., 2010), GFP-positive DFCs at the leading edge frequently generate filopodium (arrow) and lamellipodium (bracket) (right panels). These findings suggest that, in addition to the passive migration through the formation of DFC-EVL tight junction, DFCs have a potential to migrate toward the vegetal pole.

During epiboly (6–10 hpf), the EVL cells tightly bind to the yolk syncytial layer (YSL) and purse string contraction of the actin in YSL drives the migration of EVL toward the vegetal pole (Lepage and Bruce, 2010; Lee, 2014). Because a subset of DFCs is linked to EVL through the formation of tight junction, it has been suggested that DFC-EVL tight junction is important to mediate the vegetal migration of DFCs during epiboly (Figures 1, 2). However, live imaging has revealed that DFCs located at the leading edge side of the cluster frequently generate cell protrusions such as filopodia and lamellipodia toward the vegetal pole (Ablooglu et al., 2010 and see also Figure 2). These findings suggest that, in addition to passive migration mediated by DFC-EVL tight junction, DFCs have the potential to migrate themselves toward the vegetal pole.

In collective cell migration of endothelial cells, the cells behave dynamically. For instance, a follower cell sometimes moves to the leading edge and becomes a leader cell (Jakobsson et al., 2010; Arima et al., 2011; Rorth, 2012), suggesting that cell behaviors are highly variable in individuals and positions, and that cell identities as leaders and followers can be changeable during collective cell migration. If individual DFCs behave dynamically during collective DFC migration, signal activities, which regulate cell collectivity and/or migration, are different among individual DFCs and change from time to time even in an individual DFC. Therefore, precise observation of individual DFC behaviors, adhesive properties, and signal activities in living zebrafish embryos will be important to understand mechanisms of collective DFC migration.

## Conclusion and outlook

Many studies using zebrafish as a model system of vertebrate development have provided us with new insights into how the laterality organ (KV) breaks left-right symmetry. Focusing on cell collectivity regulation during KV organogenesis, we now understand the importance and the regulatory mechanism of cell collectivity in these processes. Furthermore, we show that cell adhesive properties change during collective DFC migration, indicating that multicellular tissues/organs are more dynamic than previously thought. Despite this substantial progress, many important questions remain. For instance, how do collective cell dynamics contribute to generating functional organs? How does the pairing of tight junction change? Are adherens junctions, tight junctions, and cell-ECM interaction coordinated? Does mechanical force contribute to collective DFC migration? Does collective DFC migration have analogy with other collective cell migrations seen in normal development, wound repair, and cancer invasion? It is of great interest to fill in these gaps to further clarify the regulatory mechanisms and importance of collective cell migration during organogenesis.

### Conflict of interest statement

The authors declare that the research was conducted in the absence of any commercial or financial relationships that could be construed as a potential conflict of interest.
